# Subclinical Hypothyroidism: Frequency, clinical presentations and treatment indications

**DOI:** 10.12669/pjms.334.12921

**Published:** 2017

**Authors:** Mubashir Alam Khan, Tasnim Ahsan, Urooj Lal Rehman, Rukhshanda Jabeen, Saad Farouq

**Affiliations:** 1 Dr. Mubashir Alam Khan, MBBS, FCPS. Endocrine Fellow, Medical Unit II, Jinnah Post Graduate Medical Centre, Karachi, Pakistan; 2 Prof. Tasnim Ahsan, MBBS, MRCP, FRCP, FRCP, FRCP, FCPS. Retired Professor and Head of Medicine, Endocrinology & Metabolism & Diabetes; 3 Dr. Urooj Lal Rehman, MBBS, MCPS, FCPS. Assistant Professor, Endocrine Fellow, Medical Unit II, Jinnah Post Graduate Medical Centre, Karachi, Pakistan; 4 Dr. RukhshandaJ abeen, MBBS, FCPS, Assistant Professor, Endocrine Fellow, Medical Unit II, Jinnah Post Graduate Medical Centre, Karachi, Pakistan; 5 Saad Farooq, MBBS year IV student, Aga Khan University Hospital, Karachi

**Keywords:** Subclinical Hypothyroidism, High TSH

## Abstract

**Objective:**

To determine the frequency, modes of clinical presentation and indications for replacement therapy in a cohort of patients with subclinical hypothyroidism (SCH).

**Methods:**

This study was conducted at the Endocrine and Diabetes Unit of Jinnah Postgraduate Medical Centre from September 2007 – October 2015. This was a retrospective chart analysis of prospectively collected data in whichthe medical records of 4448 patients who had presented to the Endocrine Clinic from 2007 to 2015 were reviewed. A total of 2760 (62.05%) patients were diagnosed withthyroid disorders, whereas 260 (9.42%) patients had SCH. The SCH patients were between the age of 12 to 70 years; TSH was> 4mIU/l with normal levels of FT3 and FT4. Patients were enrolled using a predesigned structured proforma. Those having chronic systemic diseases were excluded from this study. SPSS 13 was used to evaluate the data.

**Results:**

Femalepatients comprised93.8% (244 patients) of those with SCH, whereas only 6.2% (16 patients) were male. Common presenting symptoms were, lethargy in 146 patients (56.2%); increase in weight in 102 patients (39.2%) and menstrual irregularities in 90 patients (34.6%). TSH level of < 10mIU/l (4 - 10) was seen in 177 patients (68.1%) and 83 patients (31.9%) had TSH ≥ 10mU/l. Thyroxine was given to 183 (70.4%) of these patients. Common treatment indications were TSH of ≥ 10, which was seen in 83 patients (31.9%), subfertility in 32 patients (12.3%), troublesome symptoms suggestive of hypothyroidism in 31 patients (11.9%) and high titers of antibodies in 23 patients (8.8%).

**Conclusion:**

SCH is frequently seen in our population, with most patients complaining of lethargy. The most common treatment indications were a TSH ≥ 10mIU/l, whereas troublesome symptoms of hypothyroidism and subfertility were the common treatment indications in patients who had a TSH of < 10mIU/l.

## INTRODUCTION

Subclinical hypothyroidism (SCH) is a disorder of the thyroid gland characterized by elevated TSH and normal FT3 and FT4. Since clinical presentation is so varied, the only way to diagnose this condition is through biochemical testing. Causes are similar to those of overt hypothyroidism; most common being chronic autoimmune thyroiditis associated with anti-thyroid peroxidase antibodies (Hashimoto’s thyroiditis), whereas others include sub-acute thyroiditis, post-partum thyroiditis, previous hyperthyroidism, in association with other autoimmune diseases, thyroid injury/inflammation due to radiation, surgery, medication and thyroid infiltration.^1^

The prevalence of SCH is reported to be around 4-10% in the adult population, however this varies with different populations, with more cases occurring in iodine sufficient areas.^2^-^4^ The prevalence is even higher in people taking thyroid medications.^5^ Like other thyroid disorders, SCH is also much more common in women as compared to men and increases with age. Around 2-5% of SCH patients are likely to progress to overt hypothyroidism every year.^5^,^6^

Generally, there are two categories of SCH according to the elevation in serum TSH level; slightly increased TSH levels (4.0–10.0mIU/l), and severely increased TSH value (>10mIU/l), but the lower limit of TSH that should be used is still controversial with many studies using different cut offs. Almost 90% of patients with SCH have milder levels of increased TSH (4-10mIU/l).^7^,^8^

Consequences of SCH include increased risk of cardiovascular disease such as coronary artery disease, dyslipidemia, liver disease, neuropsychiatric symptoms and it may lead to subfertility, low birth weight and miscarriages. Treatment of SCH with mildly increased TSH is controversial with many studies reporting no benefit with treatment, whereas substantially increased TSH is often treated by commencing thyroid hormone replacement.^9^

Clinically, individuals are quite often asymptomatic but manifestations may include non-specific complaints or symptoms similar to those seen in overt hypothyroidism; the most frequent symptoms reported are memory impairment, slowness of thinking, muscle cramps, muscle weakness, tiredness, dry skin, feeling colder, hoarseness of voice, puffy eyes and constipation.^2^

Hypothyroidism has been associated with altered ovulatory function, menstrual irregularities, subfertility, and higher recurrent miscarriage rates. In a study of subfertile women planning an in vitro fertilization cycle, TSH levels have been shown to be significantly higher among those who produced oocytes but failed to be fertilized.^10^

Several studies have investigated the relationship between SCH and lipid abnormalities and have found that SCH is associated with high triglycerides, total cholesterol and LDL cholesterol. These effects are more pronounced in patients with TSH>10mIU/l, but few studies have observed abnormal lipids in patients with mild-SCH.^2^,^11^,^12^ The increased risk of CHD has also been associated with mild-SCH in a recent meta-analysis of observational studies, but the risk was more in patients having TSH>10mIU/l.^13^

Recent studies have reported an increased risk of gestational diabetes and pre-eclampsia in pregnant females with SCH, compared with euthyroid population.^14^,^15^ Current evidence indicates that even mildly raised TSH was associated with increased risk of miscarriage and foetal death.^16^ The current guidelines suggest treating SCH with levothyroxine before conception and during gestation with the aim of keeping TSH within trimester-specific reference range.^16^ Ourobjective was to determine the frequency, modes of clinical presentation and indications for replacement therapy in a cohort of patients with subclinical hypothyroidism (SCH).

## METHODS

This observational study was a retrospective chart analysis of prospectively collected data at the Endocrine and Diabetes Unit of Jinnah Postgraduate Medical Centre (JPMC) from 2007–2015. JPMC is a tertiary care public sector institution in Karachi, Pakistan. Approval was taken from the Institutional Ethical Review Committee before starting the study. All the patients who had increased TSH (>4mIU/l) with normal FT3 and FT4, were between the ages of 12 and 70 years were included in this study. They were grouped according to age into 3 groups; 12-30, 31-50 and 51-70 years. Patients were classified into two categories according to serum TSH level: mildly increased TSH levels (4.0–10.0mIU/l) and moderately increased serum TSH concentrations (≥10.0mIU/l). The results were interpreted as frequencies and percentages. All patients who had raised TSH with chronic systemic diseases, thyroid hormone intake or previous history of thyroid disease, were excluded from the study. A detailed history and general physical examination was done. Diagnostic algorithms were based on 2013 guidelines from European Thyroid Association.^9^ Measurements of TSH, FT3 and FT4 were done in all patients, whereas thyroid antibodies (anti-thyroid peroxidase antibodies and anti-thyroglobulin antibodies) could only be done in 118 patients due to cost constraints. These tests were performed on automated immunoassay analyzers (GENESYS 5000 Series Multi-well Gamma Counters) with standard prescribed procedures.

All patients who had a TSH of ≥10mIU/l were treated with thyroxine, whereas patients who had a TSH of<10mIU/l were treated only if they had deranged lipid profile, subfertility in females, symptoms like constipation and chronic fatigue, high titers of antibodies, associated medical conditions, like alopecia universalis or pregnancy. Patients who had these treatment indications were treated with levothyroxine. Data was analyzed using SPSS software version 13 (IBM company, Chicago, IL, USA).

## RESULTS

A total of 260 patients were included in this study. The mean age was 30.73±11.93 years; 244(93.8%) patients were female and 16(6.2%) were male. Patients presented with varied signs and symptoms, more common being lethargy (56%), goitre (49.2%), palpitation (46.9%), muscle weakness (42.7%) and weight gain (39.2%).(Table-I)

**Table-I:** Signs and symptoms of Subclinical Hypothyroidism.

		*Total (n=260)*	*Female (n=244)*	*Male (n=16)*
Lethargy		146(56.2%)	137(56.1%)	9(56.3%)
Goitre		128(49.2%)	123(50.4%)	5(31.3%)
Palpitation		122(46.9%)	119(48.8%)	3(18.8%)
Muscle weakness		111(42.7%)	106(43.4%)	5(31.3%)
Change in weight	Increased	102(39.2%)	95(38.9%)	7(43.8%)
	Decreased	61(23.5%)	58(23.8%)	3(18.8%)
Heat Intolerance		97(37.3%)	94(38.5%)	3(18.8%)
Change in Bowel Habits	Increased	15(5.8%)	15(6.1%)	0(0%)
	Decreased	85(32.7%)	80(32.8%)	5(31.3%)
Cold Intolerance		84(32.3%)	78(32%)	6(37.5%)
Change in voice		72(27.7%)	67(27.5%)	5(31.3%)
Appetite	Increased	38(14.6%)	36(14.8%)	2(12.5%)
	Decreased	69(26.5%)	65(26.6%)	4(25%)
Tremors		48(18.5%)	45(18.4%)	3(18.8%)
Anxiety		27(10.4%)	25(10.2%)	2(12.5%)

The Mean TSH was 10.4±7.77mU/l, with a minimum value of 4.14mU/l and maximum of 50mU/l. The ratio of female and male patients with TSH<10 were almost equal 68% and 68.8% respectively. Around 70% of patients aged < 50 had a TSH<10, whereas in patients aged >50 years, 64% had a TSH of ≥10mU/l.

Thyroid antibodies could be done in only 118(45.4%) patients due to cost constraints; 84(71.2%) of these patients were positive for antibodies results. (Table-II)

**Table-II:** Laboratory Investigations of the study Population.

*TSH*				

		*Total (n=260)*	*Female (n=244)*	*Male (n=16)*
TSH	<10	177(68.1%)	166(68%)	11(68.8%)
	≥10	83(31.9%)	78(32%)	5(31.2%)

***Antibodies***				

		***Total (n=118)***	***Female (n=112)***	***Male (n=6)***

Antibodies	TPO+ve	21(17.8%)	21(18.75%)	0(0%)
	Tg+ve	7(5.93%)	6(5.35%)	1(16.66%)
	Both+ve	56(47.5%)	54(48.21%)	2(33.33%)
	Both–ve	34(28.8%)	31(27.68%)	3(50%)

***Antibodies vs TSH***				

		***Total (n=92)***	***Female (n=88)***	***Male (n=4)***

TSH<10	Positive Antibodies	64(69.57%)	63(71.59%)	1(25%)
	Negative Antibodies	28(30.43%)	25(28.41%)	3(75%)
		Total(n=26)	Female(n=24)	Male(n=2)
TSH≥10	Positive Antibodies	20(76.92%)	18(75%)	2(100%)
	Negative Antibodies	6(23.08%)	6(25%)	0(0%)

Of the total patients, 183(70.4%) received treatment with thyroxine. Most common treatment indication in both male and female patients was a TSH≥10 and troublesome symptoms of hypothyroidism. All subfertility patients were female. No male patient was started treatment because of high titre of antibodies.(Table-III)

**Table-III:** Indications for Treatment of Sub clinical Hypothyroidism.

*Total Patients Treated*			

	*Total (n=260)*	*Female (n=244)*	*Male (n=16)*
Treated Patients	183(70.4%)	174(71.31%)	9(56.25%)
Untreated Patients	77(29.6%)	70(28.68%)	7(43.75%)

***Treatment Indications***			

	***Total (n=183)***	***Female (n=174)***	***Male (n=9)***

High TSH(≥10)	83(45.35%)	78(44.83%)	5(55.55%)
Subfertility	32(17.48%)	32(18.39%)	0(0%)
Symptomatic	31(16.94%)	28(16.09%)	3(33.33%)
Positive Antibodies	23(12.57%)	23(13.22%)	0(0%)
Deranged Lipid	1(6%)	10(5.75%)	1(11.11%)
Profile	1		
Other disease*	3(1.64%)	3(1.72%)	0(0%)

* Two patients were pregnant and one had alopecia totalis.

## DISCUSSION

The prevalence of SCH in Pakistan has been reported as 13.6% in females and 9.2% in males from a cohort from Karachi, whereas the frequency of this disorder in our hospital based study population was 9.4%.^17^ SCH predominantly affected female patients in this cohort, which is consistent with virtually every major study reported on hypothyroidism prevalence. In a cohort from UK the prevalence of SCH was reported as 7.5% in women and 2.8% in men.^18^ The Colorado study found 9% of the population to be subclinically hypothyroid; among subjects taking thyroid medication an even larger percentage (17.6%) were subclinically hypothyroid.^2^ In the National Health and Nutrition Examination Survey (NHANES), a lower prevalence of around 4.3% was seen.^7^ Higher levels of TSH were observed with increasing age in this study cohort as reported earlier also.^2^

In a study investigating the link of iodine with thyroid disorders, it was found that SCH is much more common in iodine sufficient areas (23.4%), as compared to iodine deficiency areas(4.2%). However, goiter was seen in 39% of the subjects from iodine deficient areas and only 12.2 % of the subjects in iodine sufficient areas.^4^

An overwhelming majority of females (72%), in those tested for thyroid antibody, were positive in this study which is in accord with the NHANES III study.^7^ The Whickham survey reported that a twofold rise in serum TSH would increase the probability of developing overt hypothyroidism from 1to 4%; this risk further increased to 38% if positive for TPO antibodies. These results suggest that if a population is followed, a significant number of SCH cases may also develop overt hypothyroidism.^3^ The presence or absence of antibodies has not been shown to have a significant impact on the emergence of SCH, therefore routine measurement of thyroid antibodies is not recommended.^5^ The highest percentage of women with antibody positivity were in the group who had a TSH between 5 and ≤10mIU/l in a study done in pregnant women, which is in contradiction to our results, whereby more patients who had a TSH≥10 had positive antibodies also.^19^

The Rotterdam Study reported SCH being an independent risk factor for myocardial infarction (MI) and atherosclerosis in elderly women, especially if they are positive for anti-TPO antibodies.^20^ An increased cardiovascular risk has been reported with TSH<10mIU/l in younger adult population (<70 years)^21^; it is suggested that levothyroxine replacement therapy in patients with SCH was associated with less CHD risk in these younger subjects.^22^

Treatment of SCH is still controversial. If untreated, patients might develop cardiac dysfunction, dyslipidemias, symptoms of hypothyroidism or progression to overt hypothyroidism and neuropsychiatric symptoms, but this has not yet been conclusively proven. On the other hand, if treatment is started when it is not indicated, patients may develop subclinical hyperthyroidism.^5^

A large questionnaire-based study on 25,862 patients showed a significant difference, although small, in symptoms between euthyroid and SCH patients.^2^,^9^ There is also some evidence of benefits regarding symptom improvement (especially tiredness) with levothyroxine treatment inpatients having TSH<10mIU/l.^23^

Most often therapy is started when TSH exceeds 10mIU/l or there are symptoms of hypothyroidism, therefore clinical context is central to the decision to institute replacement therapy. In our study also most of the patients who were started on thyroxine therapy were because of TSH≥10 and symptoms of hypothyroidism.

In clinical practice guidelines for hypothyroidism in adults, the American Association of Clinical Endocrinologists and the American Thyroid Association have recommended treatment with L-thyroxine be considered in women of childbearing age with SCH when they are planning a pregnancy.^24^ Two small randomized trials found that the miscarriage rate was significantly lower in the L-thyroxine group than in the placebo group, while the clinical pregnancy rate and delivery rate were both significantly higher.^25^,^26^

Similarly, trials have observed heterogeneous effects of levothyroxine therapy on lipid profile in patients having SCH. Although effects are more pronounced in patients with initial TSH>10mIU/l, few trials have also revealed significant improvements in lipid profile after levothyroxine treatment in patients with TSH<10mIU/l, thus reducing cardiovascular risk.^23^,^27^

## CONCLUSION

SCH is frequently seen in our population, but is often asymptomatic or there are vague symptoms leading to the diagnosis being missed. Patients may present with symptoms similar to hypothyroidism and this warrants a full thyroid check-up. Treatment should be started if patients have a TSH≥10 and/or troublesome symptoms of hypothyroidism, which decrease quality of life. The need for thyroxine therapy in pregnant woman with SCH is fairly well established. We recommend screening in patients older than 50 years of age due to the high incidence in this age group and potential adverse side effects resulting from not treating this condition.

### Authors’ contributions

**MAK:** Data collection, Statistical analysis. Preparation of manuscript.

**MAK, TA, ULR, RJ and SF:** Concept, data collection, statistical analysis.

**TA:** Editing and Supervision of the study.
